# Differential Role for CD80 and CD86 in the Regulation of the Innate Immune Response in Murine Polymicrobial Sepsis

**DOI:** 10.1371/journal.pone.0006600

**Published:** 2009-08-12

**Authors:** Anna Nolan, Hiroshi Kobayashi, Bushra Naveed, Ann Kelly, Yoshihiko Hoshino, Satomi Hoshino, Matthew R. Karulf, William N. Rom, Michael D. Weiden, Jeffrey A. Gold

**Affiliations:** 1 Division of Pulmonary/Critical Care, New York University, School of Medicine, New York, New York, United States of America; 2 Division of Pulmonary/Critical Care, Oregon Health and Science University, Portland, Oregon, United States of America; New York University School of Medicine, United States of America

## Abstract

**Background:**

Inflammation in the early stages of sepsis is governed by the innate immune response. Costimulatory molecules are a receptor/ligand class of molecules capable of regulation of inflammation in innate immunity via macrophage/neutrophil contact. We recently described that CD80/86 ligation is required for maximal macrophage activation and CD80/86^−/−^ mice display reduced mortality and inflammatory cytokine production after cecal ligation and puncture (CLP). However, these data also demonstrate differential regulation of CD80 and CD86 expression in sepsis, suggesting a divergent role for these receptors. Therefore, the goal of this study was to determine the individual contribution of CD80/86 family members in regulating inflammation in sepsis.

**Methodology/Principal Findings:**

CD80^−/−^ mice had improved survival after CLP when compared to WT or CD86^−/−^ mice. This was associated with preferential attenuation of inflammatory cytokine production in CD80^−/−^ mice. Results were confirmed with pharmacologic blockade, with anti-CD80 mAb rescuing mice when administered before or after CLP. *In vitro*, activation of macrophages with neutrophil lipid rafts caused selective disassociation of IRAK-M, a negative regulator of NF-κB signaling from CD80; providing a mechanism for preferential regulation of cytokine production by CD80. Finally, in humans, upregulation of CD80 and loss of constitutive CD86 expression on monocytes was associated with higher severity of illness and inflammation confirming the findings in our mouse model.

**Conclusions:**

In conclusion, our data describe a differential role for CD80 and CD86 in regulation of inflammation in the innate immune response to sepsis. Future therapeutic strategies for blockade of the CD80/86 system in sepsis should focus on direct inhibition of CD80.

## Introduction

Sepsis, the systemic inflammatory response to infection (SIRS), is a devastating condition affecting nearly 750,000 people/year and resulting in over $17billion/year in health care expenditure [1], [2]. Currently, sepsis is the leading cause of death in the ICU and 10^th^ leading cause of death overall [3]. However, in spite of maximal supportive care and appropriate antimicrobial therapy, mortality remains in excess of 25% underscoring the need for better adjuvant therapies [1], [2].

The innate immune response forms the corner stone of regulation of inflammation and pathogen control in sepsis. This is characterized by an initial burst of pro-inflammatory cytokines, such as IL-6, TNF-α and IL-1β, which in controlled settings, can recapitulate many of the clinical findings of sepsis. However, numerous trials have shown that neutralization of any of these cytokines individually has little to no impact on survival [4], [5]. One potential reason for their failure is the redundant and overlapping nature of many of these individual cytokines [6]. For example, while neutralization of ether TNF-α or IL-1β has little effect on mortality in humans or in mice subjected to lethal polymicrobial sepsis via Cecal Ligation and Puncture (CLP), combined neutralization does improve survival in CLP [7], [8], [9]. As a result, many investigators have begun to target receptors/mediators capable of simultaneously regulating numerous pro-inflammatory cytokines.

Costimulatory molecules are one class of receptors which have recently been implicated as fulfilling this role in the innate immune response [10], [11], [12], [13]. CD80 and CD86 represent one class of costimulatory receptors. They are stimulated via CD28 while CTLA4 serves as both a stoichiometric inhibitor of CD28-CD80/86 engagement as well as serving to directly induce immunosuppressive signals within dendritic cells [14], [15], [16]. Given the high degree of homology and functional overlap between CD80 and CD86, studies investigating their function have sought to either inhibit their common ligand (CD28) or to use a CD80/CD86^−/−^ mouse.

While a large body of evidence points to a critical role for the CD28-CD80/86 system in regulating inflammation in autoimmune disease and graft vs. host disease in the adaptive immune response, our group and others have now described a similar role in the innate immune response. Specifically, neutrophils expressing CD28 activate macrophages in a contact dependent manner via engagement of CD80/86 [17]. In turn, CD80/86 signal via NF-κB to induce numerous cytokines, most notable IL-6 [14]. *In vivo*, deletion or blockade of CD80/86 improves survival and attenuates pro-inflammatory cytokine production in CLP [18].

However, these studies also demonstrated that monocyte expression of CD80 and CD86 were differentially regulated in sepsis [18]. Specifically, sepsis was associated with an increase in CD80 expression, while there appeared to be a downregulation of constitutive CD86 expression. Further, recent studies suggest CD80 and CD86 have differential regulation in the inflammatory response *in vivo* in diseases regulated by the adaptive immune response, including allergic rhinitis and graft rejection [19], [20]. Combined, these data imply a possible differential role for CD80 and CD86 in regulating mortality and inflammation in the innate immune response as well. Consequently, the goals of this study were to determine the individual contribution of CD28, CD80 and CD86 to the inflammatory response in murine sepsis, and to better correlate expression of these molecules with outcome in humans with sepsis and septic shock.

## Methods

### Ethics Statement

All animal studies were approved by the New York University and Oregon Health and Sciences University Institutional Animal Care & Use Committee (IACUC). For human studies, the protocol was approved by the Oregon Health and Sciences University Institution Review Board and written consent was obtained from all subjects or their designated representative.

### Mice

6–8 week old female C57BL/6 (WT), CD28^−/−^, CD80^−/−^ and CD86^−/−^ mice were purchased from Jackson Labs. All mice were on a C57BL/6 background. Mice were allowed to acclimatize for 1 week prior to use. All mice were housed in SPF facility. **Cecal Ligation and Puncture**. CLP was performed as previously described [10], [11], [12], [13]. Briefly, mice were anesthetized with 2.5% isoflourane and underwent CLP with a 19 gauge needle. Mice received 1 cc 0.9% saline subcutaneously for resuscitation. At specified times, the mice were used to collect plasma, bronchoalveolar lavage (BAL) and peritoneal lavage (PL) (3 cc) as previously described [10]. For survival experiments, mice were monitored for a total of 14 days. For antibody inhibition, 250 µg of α-CD80 (16-10A1; hybridoma from ATCC) or α-CD86 (GL-1, hybridoma from ATCC) were injected *ip* 4 hrs prior to or 2 hrs post -surgery. Rat IgG (Biolegend) was used as an antibody control and PBS as a vehicle control. As there was no difference between the 2, these groups were combined where indicated. IL-6, IL-10, IL-12p40, and IL-1β were determined by commercially available immunoassays (R&D systems) and performed according to the manufacturers' specifications.

#### Flow Cytometry

Flow cytometry was performed as previously described [18]. Whole blood or peritoneal lavage were collected and 1×10^6^ cells were then labeled with the following antibodies: CD80, CD86, CD14 at optimal concentration for 45 min in the dark. Red blood cells were lysed with RBC lysis buffer then the cells were fixed with 0.1%paraformaldehyde and analyzed on a BD LSRII 8-color analyzer with FloJo software. All reagents were purchased from BDPharmigen. BD compensation beads were used to calibrate the instrument before each use. PMNs were identified by FSC/SSC characteristics; mononuclear cells by FSC/SSC, CD11b^+^ and CD14^+^, Isotype antibody labeled cells were used to control for nonspecific staining.

#### Purification of PMN and preparation of Lipid rafts

Human PMNs and lipids rafts were obtained from healthy volunteers and prepared as previously described [17]. Briefly, PMN were isolated from whole blood by Ficoll-Hypaque (Amersham Pharmacia) sedimentation. PMN (>99.9% granulocytes, >95% PMNs) were separated by dextran sedimentation (3% dextran in 0.9% NaCl solution @ 1 *g*, 30 min). PMN were activated by 100 ng/ml LPS (Sigma). For Detergent Resistant Membrane (DRM)/Lipid raft isolation, cells were lysed on ice with 200 µl of 1% Triton X-100 and gently mixed with an equal volume of 80% sucrose (wt/vol) and placed in the bottom of a Beckman centrifuge tube. The sample was then overlaid with 1000 µl of 30% sucrose and 600 µl of 5% sucrose spun for 16–24 h at 44,000 rpm at 4°C in a Beckman TLS 55 swing rotor using Beckman OPTIMA TLX Ultracentrifuge. 200 µl fractions were harvested serially from the top of the gradient. The DRM/raft fraction was obtained in fractions 1–4.

### Immunoprecipitation

Human monocytes derived macrophages were isolated from healthy volunteers and prepared as previously described [17].Cells harvested and washed with PBS ×3 and total cell lysate prepared using NP40 lysis buffer as previously described. Lysate (normalized for equal protein content) was then incubated with 10 µg antibodies to CD80 or CD86. One ml of extract was then incubated with end-over-end mixing for 2 hours at 4°C. Fifty µl of Protein A/G Sepharose (Santa Cruz Biotech) were washed with 1 ml of lysis buffer. This was repeated 3 times and then excess buffer was carefully removed. After the 2 hour incubation, the washed Protein A/G Sepharose was added to the immunoprecipitates and mixed for 1 hour at 4°C. The immunoprecipitates were washed 3X with 1 ml of lysis buffer. Samples then assayed by Western blot as previously described.

### Immunofluorescence Confocal Laser Microscopy

Cells were attached to poly-L-Lysine-coated coverslips and fixed in 4% paraformaldehyde/PBS followed by acetone/methanol (1∶1, vol./vol.). After blocking in non-specific IgG for 30 minutes, they were incubated with primary antibody (1∶1000) over night. Following washing in PBS, cells were incubated with appropriate secondary antibodies containing Alexa-Fluor-labeled 488, 563, or 633 IgG (H+L) antibodies (Molecular Probes, OR) (1∶500) for 30 minutes as previously described [17]. After final wash, samples were mounted with VectraShield mounting medium (Vector Laboratories) and images were obtained and quantified by Leica TCS NT imaging software. Images were processed with Photoshop (Adobe Systems, Mountain View, CA).

### Human studies

All patients meeting SCCM/ACCP criteria for sepsis in the first 24 hrs of ICU stay were eligible for inclusion [21]. Patients were excluded for the following reasons; presence of a Do Not Resuscitate order or decision to institute comfort care measures, Hgb<7 g/dl or the presence of active bleeding requiring more than 2 units of packed red blood cells. After obtaining informed consent, 25 cc of blood was collected into glass (serum) or EDTA coated tubes (platelet poor plasma) within the first 24 hrs of admission. Clinical data including APACHE II scores were recorded at the time of the blood draw. Patients were followed for 28 days or until death or hospital discharge.

### Statistics

Survival was analyzed by Kaplan-Meier analysis. All comparisons between groups were performed by Mann-Whitney t-test (2 groups) or ANOVA (multiple groups). Correlations were made with Spearman's test. All statistics were done with GraphPad Prism 5.0 (San Diego, CA).

## Results

**Figure 1:**
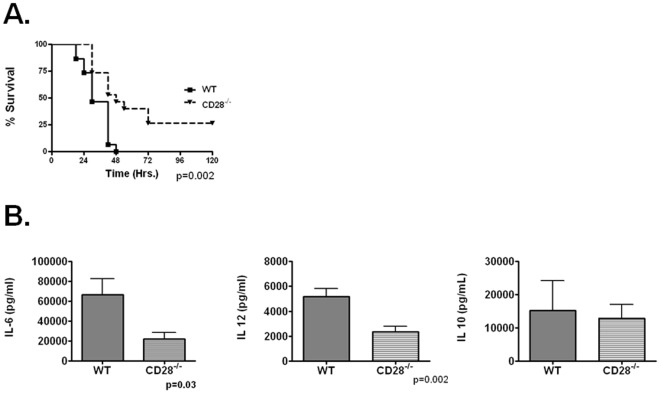
CD28^−/−^ mice are protected from lethality of polymicrobial sepsis. Panel A-WT (n = 15) and CD28^−/−^ (N = 15) underwent CLP and were monitored for survival. Panel B. WT and CD28^−/−^ mice underwent CLP and were sacrificed at 18 hrs and plasma collected for cytokine analysis via commercially available ELISA. N = 5/group.

We first wished to establish that CD28 was serving as the predominant ligand for CD80 or CD86 engagement in sepsis. CD28^−/−^ mice had improved overall and median survival compared to WT mice after CLP (Figure 1A). Improvement in survival was associated with attenuation in plasma IL-12 and IL-6, with little change in IL-10 (Figure 1B) or IL-1β (not shown) 18 hrs after CLP. IFN-γ, another cytokine regulated by CD28, was undetectable in both groups of animals [14]. We observed similar results in the lung (not shown), suggesting CD28 regulates inflammation in multiple tissue compartments. Together suggesting CD28, through either CD80 or CD86 is capable of mediating the inflammatory response and lethality of polymicrobial sepsis.

**Figure 2:**
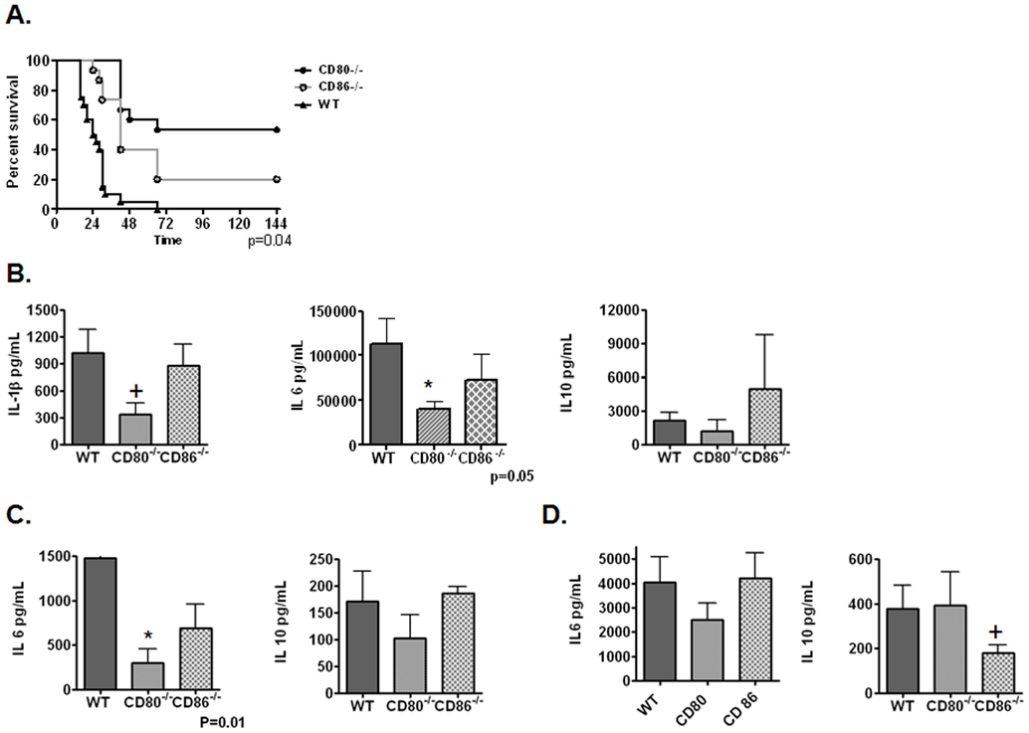
CD80 preferentially control lethality and inflammation in CLP. Panel A-WT (n = 20) and CD80^−/−^ (N = 15) and CD86^−/−^ (N = 15) underwent CLP and were monitored for survival. P<0.04 (CD80^−/−^ vs. CD86^−/−^), p<0.001 (CD80^−/−^ vs. WT) and p<0.01 (WT vs. CD86^−/−^). Panel B–D. WT and CD80^−/−^ and CD86^−/−^ mice underwent CLP and were sacrificed at 18 hrs and plasma (Panel B), BALF (Panel C) and PL (Panel D) were collected for cytokine analysis via commercially available ELISA. N = 8–10 mice/group. + = <0.1, * = <0.05.

We next sought to determine the relative contributions of CD80 and CD86 to mortality and inflammatory cytokine production in polymicrobial sepsis. Interestingly, CD80^−/−^ mice had a marked improvement in median and overall survival compared to WT (p<0.001) and CD86^−/−^ mice (p = 0.01). In contrast, there was only a modest benefit observed in median survival in CD86^−/−^ mice compared to WT controls (median survival 42 vs. 25 hrs; p = 0.002) with no change in absolute survival (20% vs. 5%; p>0.2) (Figure 2A). This was associated with a preferential attenuation of plasma IL-6 and IL-1β in CD80^−/−^ mice compared to WT or CD86^−/−^ mice (Figure 2B). We observed similar results in BALF (Figure 2C). However, in peritoneal lavage, IL-10 was preferentially attenuated in CD86^−/−^ mice (Figure 2D). This being more consistent with some reports describing a preferential role for CD86 in regulating IL-10 production and is consistent with our previous description of differential CD86 expression in various tissue compartments in CLP, with increased levels of CD86 found in PL compared to blood [22].

**Figure 3:**
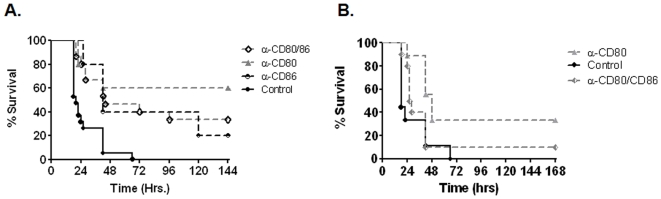
CD80 blockade preferentially improves survival after CLP. Panel A-WT C57BL/6 mice were administered either 250 µg anti-CD80, anti -CD86 or both 4 hrs prior to CLP and monitored for survival (n = 10/group). Data from anti -CD80/86 has been published previously [18]. P = 0.002 for anti-CD80 vs. control. Panel B-WT mice were administered control IgG or anti -CD80 or anti -CD80/86 (250 µg each) 2 hrs post-CLP and monitored for survival (n = 10/group). p = 0.002 for anti-CD80 vs. control.

We next used a pharmacologic approach to confirm the results with our knockout mice. Pre-treatment with anti-CD80 mAB significantly improved survival compared to either control IgG or anti-CD86 mAB (Figure 3A). Similar to observations with knockout mice, anti-CD86 modestly improved median survival compared to controls (42 vs. 18 hrs; p = 0.03), yet when combined with anti-CD80, antagonized the beneficial effects of anti-CD80. Anti-CD80 also proved to be an effective rescue therapy as post-CLP administration significantly improved survival compared to controls (Figure 3B). Similar to what we observed with pre-CLP therapy, addition of CD86 blockade antagonized the effects of anti-CD80 therapy.

**Figure 4:**
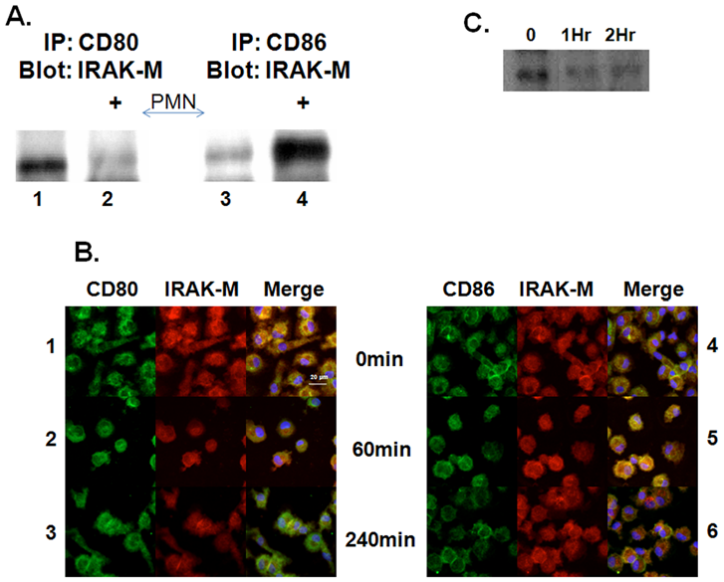
PMN membranes disassociate IRAK-M from CD80. Macrophages were treated with PMN lipid rafts for various times Panel A. Co-IP demonstrates CD80 binds IRAK-M in resting PMA differentiated THP-1 macrophages, lane 1. One hour after lipid raft treatment IRAK-M disassociates from CD80, lane 2. CD86 binds little IRAK-M in resting macrophages, lane3. There is association of IRAK-M with CD86 one hour after lipid raft addition, lane4. Panel B. Confocal microscopy of resting monocyte derived macrophages at baseline (Row 1,4) or stimulated with PMN lipid rafts for 1 hr (Row 2,5) or 4 hrs (Row 3,6). IRAK-M (Red), CD80 (Left Panel-green) or CD86 (Right Panel-green) and co-localization (Yellow). Nucelar stain with DAPI (blue). Panel C. PMA differentiated THP-1 macrophages were stimulated with PMN lipids rafts and harvested at the designated time point. Whole cell extracts were IP for TRAF-6 and blotted for IRAK-M. All lanes (panel A and C) were normalized for protein prior to immunoprecipitation.

We next sought to determine a potential mechanism for CD80 regulation of inflammation. Prior studies suggest CD28 induces NF-κB induction via CD80 and CD86 [14]. At baseline, Interleukin-1 receptor associated kinase-M (IRAK-M), a negative regulator of NF-κB signaling, was associated with CD80 as opposed to CD86. Upon addition of neutrophil lipid rafts, previously reported by our lab as a source of CD28 and stimulator of macrophages *in vitro* and *in vivo* [17], the association of CD80 with IRAK-M at 1 hr was abolished (Figure 4A). In contrast, there was an increase in association of CD86 and IRAK-M 1 hr after the addition of lipid rafts. The interaction between CD80 and IRAK-M was confirmed by immunofluorescent microscopy, which demonstrated that at baseline, there was significant co-localization of CD80 and IRAK-M. (Figure 4B, Row 1) After addition of PMN lipid rafts, while there was no change in the amount of CD80 and IRAK-M, by 4 hrs (Figure 4B Row 3), the vast majority of CD80 was no longer associated with IRAK-M (Figure 4B Row 3). In contrast, the amount of IRAK-M associated with CD86 actually increased after stimulation (Figure 4B Row 5). Finally, we confirmed that IRAK-M did indeed associate with TRAF-6 in our system and that there was a slight decrease in this association with PMN lipid raft stimulation providing another mechanism for de-repression of macrophages by PMN contact (Figure 4C).

**Figure 5:**
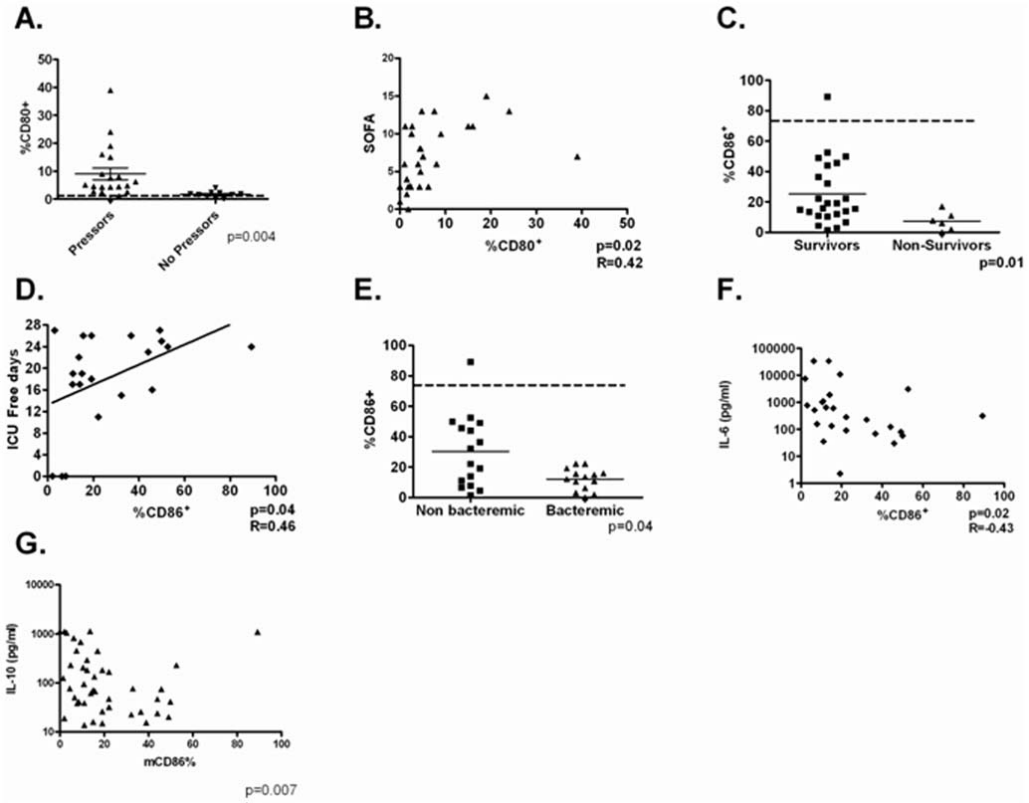
Differential Regulation of CD80 and CD86 in human sepsis. Patients with sepsis (n = 31) had blood drawn within 24 hrs of ICU admission. Samples analyzed by flow cytometry and plasma underwent cytokine analysis. Panel A-CD14^+^ monocytes expression of CD80 in subjects with (n = 20) or without (N = 10) septic shock. Panel B- Correlation between CD14^+^ monocytes expression of CD80 and SOFA Score. Panel C- CD14^+^ monocytes expression of CD86 in survivors (N = 24) and non-survivors (N = 6). Panel D- Correlation between CD14^+^ monocytes expression of CD86 and ICU Free days. Panel E-CD14^+^ monocytes expression of CD86 in subjects with (n = 16) or without (N = 14) bacteremia on presentation. Panels F and G— Correlation between CD14^+^ monocytes expression of CD86 and IL-6 (Panel F) and IL-10 (Panel G).

Finally, we sought to expand our previously reported data and determine whether differential expression of CD80 and CD86 occurred in humans with sepsis and if expression levels could serve as biomarkers for outcome [18]. Clinical characteristics of enrolled subjects are presented in Table 1. CD80, was upregulated on circulating monocytes in septic humans, with higher levels associated with both the presence of shock (Figure 5A) and correlating with overall severity of illness as determined by SOFA score (Figure 5B). There was no association between levels of CD80 and survival or cytokine levels. A relatively protective role for CD86 was supported by the presence of higher levels of expression on circulating monocytes in survivors and those with negative blood cultures (Figure 5C and E). Higher levels of CD86 also correlated with ICU free days (Figure 5D). Finally, levels of CD86 correlated inversely with circulating levels of IL-10 and IL-6 (Figure 5F and G) suggesting a relatively anti-inflammatory role for CD86 *in vivo*.

**Table 1:** Clinical Characteristics of enrolled subjects. Absolute values are presented are Mean±SEM.

	Septic Subjects (n = 40)
Age (years)	56.8±2.43
Sex (% Female)	35%
APACHE II	17.1±1.04
SOFA	6.7±0.7
Mechanical Ventilation (%)	42.5
Vasopressors (%)	65
Bactermia (%)	37.5
28 Day Mortality (%)	17.5

## Discussion

One of the major findings of this paper is the observation of improved survival in CD28^−/−^ mice after CLP. The ability of CD28 to regulate inflammation in the innate immune response to sepsis is consistent with prior reports documenting CD28 expression on PMNs and the ability of CD28, either soluble or in PMN lipid rafts, to induce NF-κB and pro-inflammatory cytokine production via CD80/CD86 [14], [17], [23]. Our results are also consistent with earlier reports suggesting an important role for CD28 in regulating abdominal abscess formation in CLP as well as the role for CD28 in mediating toxic shock in mice [6], [24]. Finally, these data support the recent observations of lethal cytokine storm in with administration of an agonist CD28 antibody in humans [25]. Together these data imply a prominent role for the CD28-CD80/86 system in regulating multiple pro-inflammatory cytokines in the innate immune response.

While CD28 is capable of activating both CD80 and CD86, the relative contribution of the interaction and differential effect of these *in vivo* has been less well defined. The finding of improved overall survival in CD80^−/−^ and not CD86^−/−^ mice implies CD80 is the dominant receptor for regulating lethality in the innate immune response in the early stages of CLP. This is corroborated by the relative attenuation in IL-6 in CD80^−/−^ vs. CD86^−/−^ mice and is consistent with the known ability of CD28 to regulate IL-6 production from dendritic cells in a CD80 dependent manner [14]. Interestingly, recent studies suggest CD86, not CD80, plays a dominant role in mediating graft vs. host disease and abdominal abscess formation [17], [19], [20]. The reasons for these contrasting results are likely due to the source of CD28 and the chronicity of inflammation. In those model systems, there is a much more prominent role for CD4^+^ T-cells, as opposed to PMNs or soluble CD28 as observed in the early phases of sepsis. Future studies are required to determine the interaction between the cellular source of CD28 and predilection for CD80 vs. CD86 activity. Finally, the improved survival with anti-CD80 as opposed to anti-CD86 confirms the results obtained with the knockout mice and the ability of anti-CD80 to rescue mice when given after the onset of sepsis suggests this is a potential therapeutic target as well.

The ability of CD80 to regulate inflammation can be in part explained by its known ability to regulate the induction of NF-κB in response to CD28 engagement. The NF-κB signaling complex contains numerous adapter molecules which serve to both stimulate and repress NF-κB signaling. IRAK-M is a well described repressor of NF-κB signaling and successful induction of pro-inflammatory signals requires loss of IRAK-M from the NF-κB signalsome [26]. We now show that IRAK-M is directly associated with CD80 and CD86 and that stimulation of macrophages with PMN lipid rafts causes preferential loss of this association with CD80, providing one potential mechanism to explain the preferential ability of CD80 to regulate multiple inflammatory cytokines. Further studies are required to ascertain whether this is specific for the presence and source of CD28 (PMNs vs. T-cells), explaining the predilection for CD80 in innate immunity while CD86 appears more prominent in the adaptive immune response.

Another potential reason for the prominent effect for CD80 in innate immunity, centers on the differential expression of CD80 and CD86 in sepsis. Our group and others have previously demonstrated that sepsis is associated with marked an increase in CD80 expression and a loss of constitutively expressed CD86 in mice [18]. We now extend these observations to humans. Similar to mice, humans with sepsis exhibit a loss of CD86 and upregulation of CD80 on circulating monocytes. A prominent role for CD80 in regulating lethal inflammation is supported by a direct correlation between CD80 levels and severity of illness (SOFA score) and presence of shock. Of even greater interest was the inverse correlation of CD86 and severity of illness, with loss of CD86 being associated with mortality, a reduction in ICU free days and increased likelihood of shock. Further, the negative association of CD86 with circulating levels of IL-10 and IL-6 suggest a potential direct anti-inflammatory role for CD86 *in vivo*. However, the mechanism of CD86 loss remains incompletely understood.

While our data provide strong evidence for a predominant role for CD80 in regulation of lethal inflammation in sepsis, the role for CD86 remains conflicted. Overall, CD86 appears to exert a protective role, especially in the context of CD80 inhibition. This is supported by the antagonistic effects of CD86 blockade/deletion on survival in the setting of CD80 blockade. A potentially beneficial/protective role for CD86 is further supported by our human observations that persistence of CD86 expression is associated with improved outcome. This finding is consistent with a prior study showing reduced levels of CD86 mRNA in lethal pediatric septic shock [27]. However, the modest survival benefit associated with isolated CD86 blockade/deletion suggests that some of these protective effects may be modulated by CD80 as well. The reason for the predilection of CD80 for lethality and CD86 for protection may lie in their relative affinities and binding kinetics for their ligands, CD28 and CTLA4, with CD80 having a relative higher affinity for CTLA4 [15], [28], [29], [30]. Thus we cannot discount a potentially protective role for a CTLA4-CD86 interaction which is unmasked by the absence of CD80 in our system. This is a distinct possibility given the ability of CTLA4 to both inhibit CD28 engagement as well as direct induce signaling, including induction of tryptophan catabolism [16]. Investigators have also described a CD28/CTLA4 independent ligand for CD86, which may also modulate our system both signaling via the receptor and ligand [31]. Addressing both of these possibilities will be the subject of further studies. Finally, it is highly probable, that the role of CD86 may indeed be tissue compartment specific. In our previous study, we noticed differential regulation of CD86 in blood (down regulation) and peritoneal lavage (upregulation) [18]. This most likely explains the differential cytokine response, especially IL-10, between these differing compartments. However, we are still unable to provide a mechanism for this differential compartment specific regulation.

However, there are many important limitations to our study. Most notably is the use of a highly lethal form of polymicrobial sepsis in our murine model. It is well established that there are multiple phases to the immune response in sepsis, with the early phases dominated by massive pro-inflammatory cytokine production, and the latter phase by immunoparalysis [2], [32]. It is likely, that during the transition to these latter stages, a more prominent role for CD86 could be observed. In addition, the mechanism for loss of CD86 expression also remains incompletely understood. Whether this results in a true loss of expression or recruitment of additional low expressing CD86 monocytes from the bone marrow is also unclear. Future studies will be required to address these questions. Finally, while our data now suggest IRAK-M may be capable of differentially regulating CD80 and CD86 mediated cellular activation, there are still multiple limitations to this data. Most notably, is we can not explain the reason for the differential affinity for CD80 and CD86 for IRAK-M under resting or stimulated conditions. Understanding the reasons for this and the true biological significance of this association will be the subject of future studies detailing all members of the NF-κB signaling complex.

In conclusion, we document a pivotal role for CD28-CD80 interaction in regulating the lethality of the acute phases of sepsis and septic shock. This occurs predominantly through the interaction between CD80 and CD28. These data suggest that any future therapies targeting this system in sepsis be directed specifically at CD80.
